# Low‐grade follicular lymphoma with interferon regulatory factor‐4 rearrangement: Expanding the spectrum of interferon regulatory factor‐4‐rearranged lymphomas

**DOI:** 10.1002/jha2.992

**Published:** 2024-08-21

**Authors:** Ali Sakhdari, Peter J. B. Sabatini, Tulasi Geevar, Tong Zhang, Jan M. A. Delabie

**Affiliations:** ^1^ Department of Laboratory Medicine and Pathobiology Laboratory Medicine and Pathobiology University Health Network Toronto Canada

**Keywords:** *IRF4* rearrangement, mutational profile, small B‐cell lymphoma

## Abstract

In recent years, the recognition of distinct lymphoma entities has expanded with advancements in molecular characterization. Large B‐cell Lymphoma with interferon regulatory factor‐4 (*IRF4*) rearrangement (LBCL‐*IRF4*‐R) is one such entity. We present a case of classic low‐grade follicular lymphoma with *IRF4* rearrangement in a 50‐year‐old male, demonstrating an unusual immunophenotypic and genetic profile reminiscent of LBCL‐*IRF4*‐R. Molecular analysis revealed mutations in *CREBBP*, *KMT2D*, *IRF4*, and *CARD11*, with previously unreported variants identified in *IRF4* and *CREBBP*. This case broadens the spectrum of B‐cell lymphomas associated with *IRF4* rearrangement by demonstrating a small B‐cell lymphoma with this genetic feature.

## 1 INTRODUCTION

In the most recent publication from the World Health Organization (WHO) Classification of Tumours of Haematopoietic and Lymphoid Tissues, one B cell lymphoma entity with interferon regulatory factor (*IRF*)‐4 rearrangement has been recognized. This entity is referred to as Large B‐cell Lymphoma with IRF4 rearrangement (LBCL‐*IRF4*‐R) [1, 2]. Histologically, LBCL‐*IRF4*‐R may manifest as grade 3 follicular lymphoma (FL‐3), diffuse large B‐cell lymphoma (DLBCL), or a combination of both, characterized by a homogeneous proliferation of centroblasts or intermediate‐size blastoid cells. LBCL‐*IRF4*‐R typically lacks a starry‐sky pattern and exhibits limited intermingling of small T cells [3]. Neoplastic cells often exhibit robust expression of IRF4/MUM1 and BCL6 with a high Ki67 index. While CD10 and BCL2 are detected in approximately 50%–60% of cases, their expression is usually partial [4, 5]. LBCL‐*IRF4*‐R is a rare subtype, primarily affecting children and young adults. It typically involves tonsillar structures and lymphoid tissues of the Waldeyer's ring [5]. While pediatric cases often respond well to high‐dose chemotherapy, adult‐onset LBCL‐*IRF4*‐R tends to be more aggressive with a less favorable prognosis [3, 6]. Recent studies have identified genetic mutations and signaling pathway alterations in LBCL‐*IRF4*‐R pathogenesis, offering potential therapeutic targets [7]. In one study, the most frequently mutated genes in LBCL‐*IRF4*‐R were *IRF4* (76%), *CARD11* (35%), and *CCND3* (24%). Mutation in *IRF4* typically showed a pattern of aberrant somatic hypermutation with predominantly an activation‐induced deaminase mutational signature [7]. In that study, *KMT2D* mutation in LBCL‐*IRF4*‐R was rare and was mostly detected in non‐*IRF4*‐R subtypes of germinal center B cells like DLBCL‐NOS.

Herein, we present a case of classic follicular lymphoma exhibiting a purely follicular growth pattern with low‐grade morphology, but with high MUM1 expression and *IRF4* rearrangement, thus broadening the spectrum of B‐cell lymphomas associated with *IRF4*‐R.

## 2 CASE PRESENTATION

The patient is a 50‐year‐old male who initially presented in October 2022 with a significant weight loss of 25 pounds. His past medical history was notable for hypertension, dyslipidemia, a 15‐pack‐year history of smoking, hepatitis B (treated with Entecavir), *Helicobacter pylori* infection, and diverticulosis. He began experiencing periumbilical pain, prompting investigation via colonoscopy and computed tomography (CT) scanning. A colonoscopy revealed colonic diverticulitis and hemorrhoids, while CT imaging of the abdomen and pelvis indicated thickening of the proximal jejunum and extensive adenopathy of the small bowel mesentery. Physical examination did not reveal palpable cervical, axillary, or inguinal lymphadenopathy or hepatosplenomegaly. A complete blood count (CBC) showed no abnormalities, with normal hepatic and renal functions. Positron‐emission tomography (PET) scan findings demonstrated moderate, abnormal fluorodeoxyglucose uptake in the loops of the proximal/mid‐small bowel, along with metabolically active mesenteric lymphadenopathy (SUV max, 6.0). No metabolically active retroperitoneal, pelvic, or other, including head and neck region, lymphadenopathy was detected. A laparoscopic excision of a small bowel lymph node was performed, and pathology confirmed the diagnosis of classic low‐grade follicular lymphoma.

## 3 HISTOLOGICAL AND IMMUNOHISTOCHEMICAL FEATURES

Microscopic examination revealed a disrupted lymph node architecture characterized by multiple, rather small nodules without polarization or tingible body macrophages. The nodules showed predominantly a monomorphic population of small atypical centrocytes, with very few large atypical cells observed consistent with grade 1 follicular lymphoma. A similar infiltrate was noted in the perinodal fatty tissue.

**FIGURE 1:**
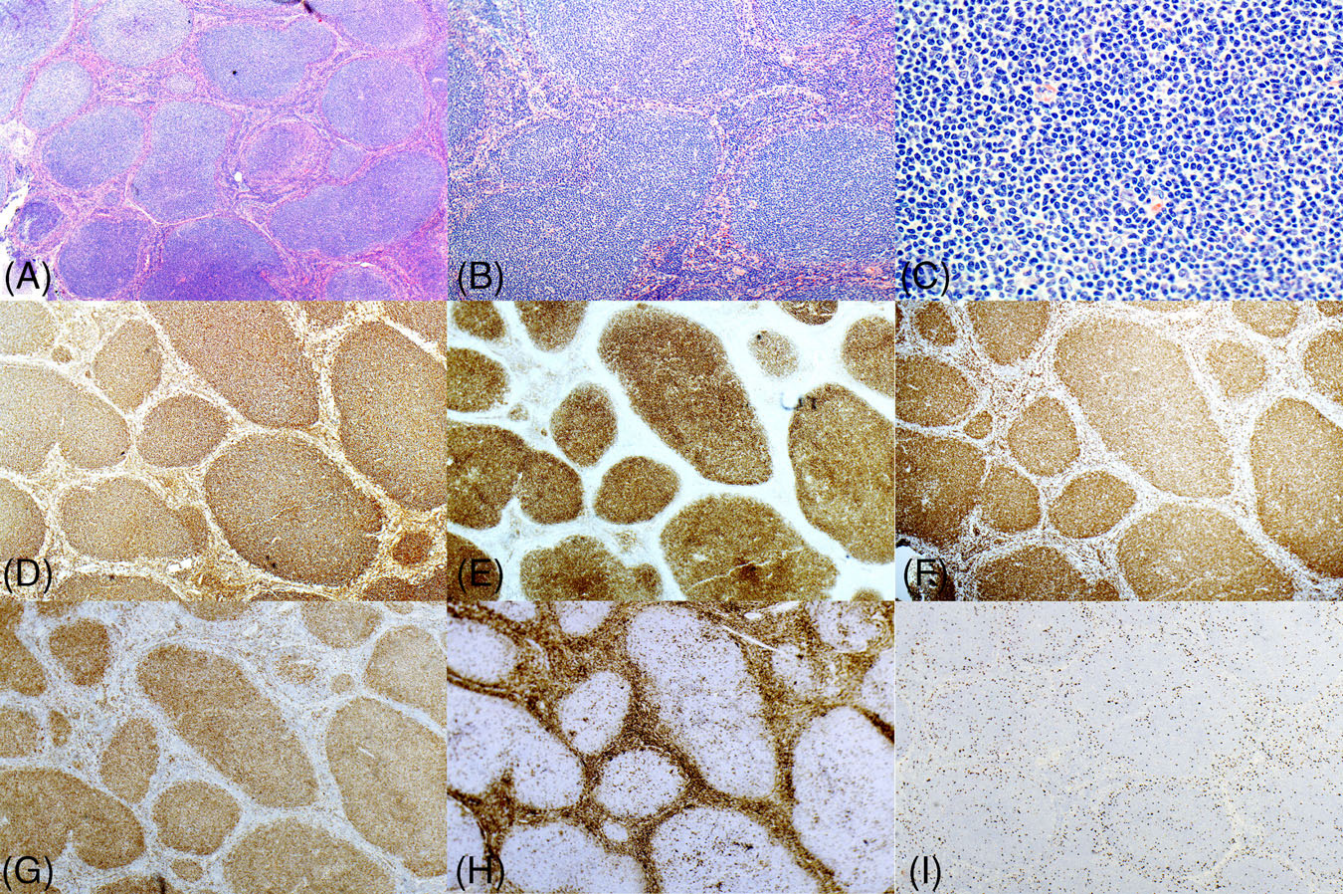
Morphological and immunophenotypic characteristics of small B cell lymphoma (SBCL) with interferon regulatory factor‐4 (*IRF4*) rearrangement. Architectural effacement of lymph node by a purely nodular lymphoma. (A–C) Hematoxylin and Eosin, 10x, 20x, and 40x) follicles are composed of sheets of small centrocytes with rare scattered centroblasts. The mantle zone is attenuated. Lymphoma cells are positive for CD20 (D, 10x), CD10 (E, 10x), BCL6, BCL2 (F, 10x), IRF4/MUM1 (G, 10x), and negative for CD3, and CD43 (H, 10x). Proliferation index is approximately 5% (I, 10x).

Immunohistochemical staining demonstrated expression of CD10, CD20, BCL2, BCL6, and MUM1 by the lymphoma cells, while they were negative for CD5, CD15, CD23, CD30, and CD43. Of note, MUM1 expression was unusually elevated for classic follicular lymphoma, and seen in more than 90% of cells. Further, the proliferation index assessed by Ki‐67 expression indicated a low index of approximately 5% (Figure 1).

Flow cytometry analysis of the same node specimen revealed 55% B lymphoid cells with aberrant immunophenotypes: CD5‐, CD10+, CD11c‐, CD19+, CD20+, CD22+, CD23‐, CD25‐, CD103‐, IGK+, and IGL‐.

## 4 CYTOGENETIC AND MOLECULAR FINDINGS

Conventional cytogenetic studies revealed no IGH::*BCL2* translocation (Vysis LSI IGH/BCL2 Dual Color Dual Fusion Probes) or *BCL6* gene (Vysis LSI BCL6 Dual Color Break Apart Rearrangement Probe) rearrangement. The absence of *BCL2* or *BCL6* rearrangements and the notably high expression of MUM1 prompted further investigation. A probe targeting the *IRF4* gene locus at 6p25.3 (Zytovision, ZytoLight SPEC IRF4/DUSP22 Dual Color Break Apart Probe) was, therefore employed. The results of this analysis demonstrated the presence of an *IRF4* rearrangement in 158 out of 200 (79%) nuclei examined.

To characterize additional genomic variants, we used a next‐generation sequencing panel enriched for genes important in B cell lymphomagenesis. Genomic DNA was extracted from formalin‐fixed paraffin‐embedded tissue using the Maxwell 16 FFPE plus LEV DNA Purification Kit (Promega). Coding regions for the selected genes (Table S1) were captured using SureSelect custom hybridization probes (Agilent) and sequenced using Illumina NextSeq500 (Illumina). The bioinformatics pipeline included alignment by the Burrows‐Wheeler Alignment (BWA‐MEM), with processing and quality metrics by GATK 3.3–0 and PICARD 1.130 and variant calling by VarScan 2.3.8. Variants were further filtered to remove those in the reference population at a frequency > 1% (gnomAD V.2.11) and contained read depths greater than 100.

Next‐generation sequencing identified mutations in *CREBBP, KMT2D, IRF4*, and *CARD11* (Table 1).

**TABLE 1:** The possible oncogenic variants detected in the case of low‐grade follicular lymphoma with interferon regulatory factor‐4 (*IRF4*)‐R.

Gene	Transcript	cDNA	Protein	Variant allele fraction (VAF)
*CARD11*	NM_032415.5	c.746A > C	p.Gln249Pro	10.50%
*CREBBP*	NM_004380.2	c.4412T > C	p.Ile1471Thr	48.10%
*IRF4*	NM_002460.3	c.213C > A	p.Phe71Leu	25.50%
*KMT2D*	NM_003482.3	c.7903C > T	p.Arg2635*	21.90%

## 5 DISCUSSION

LBCL‐*IRF4*‐R, now recognized as a distinct entity in both the International Consensus Classification and [8] the 5th edition of the WHO classification primarily affects children and young adults [1]. Its occurrence diminishes with advancing age compared to other LBCL types [9]. Typically, it presents in the head and neck region, notably the Waldeyer ring or cervical lymph nodes, and occasionally involves mesenteric lymph nodes [10]. These lymphomas predominantly belong to the germinal center B‐cell (GCB) subtype and exhibit strong expression of IRF4/MUM1 protein but lack the *BCL2* gene translocation [11]. Recent molecular analyses reveal a unique gene expression profile, including frequent mutations in IRF4 and nuclear factor kappa B (NF‐κB)‐related genes (*CARD11*, *CD79B*, and *MYD88*), affecting overexpression of downstream NF‐κB pathway genes. Although rare, LBCL‐*IRF4*‐R cases among adults display relatively more genetic complexity, with an increased mutational burden and a higher prevalence of *KMT2D* mutations compared to pediatric lymphomas [7]. Despite being a defining feature of LBCL‐*IRF4*‐R, *IRF4* rearrangement is not exclusive to this subtype, as it can also occur in other aggressive B‐cell lymphomas, particularly those associated with *MYC* or *BCL2* rearrangements [12]. The molecular analysis in our case showed variants in *CREBBP, KMT2D, IRF4*, and *CARD11*. Only the *CARD11* variant (p.Gln249Pro) and *KMT2D* (p.Arg2635*) variants have been previously reported in cancer [13, 14, 15]. Both *IRF4* and *CREBBP* variants have not been reported in the literature and further studies are needed to confirm the functional relevance of these mutations.

To the best of our knowledge, no previous cases of low‐grade classic follicular lymphoma with *IRF4* rearrangement have been reported. Herein, we present the first documented case of a 50‐year‐old male patient diagnosed with limited‐stage low‐grade classic follicular lymphoma localized in the mesenteric lymph nodes. Remarkably, this case exhibits morphological, immunophenotypic, genetic, and molecular characteristics akin to *LBCL*‐IRF4‐R. These striking similarities prompt consideration of the potential relationship between low‐grade follicular lymphoma with *IRF4* rearrangement and LBCL‐*IRF4*‐R. While it raises the intriguing possibility of a precursor relationship, the absence of a significant low‐grade follicular component in documented LBCL‐*IRF4*‐R cases, along with the lack of large cell lymphoma in our case, suggests that these may be two distinct biological entities. Notably, our patient's response to rituximab and bendamustine therapy, evidenced by complete remission on the latest PET scan post‐cycle 6x, underscores the effectiveness of conventional follicular lymphoma treatments. Recent studies have also suggested the de‐escalation of conventional chemotherapy for LBCL‐IRF4, as many cases exhibit an excellent prognosis [11]. The mutational profile of the current case closely resembles conventional BCL2‐rearranged low‐grade follicular lymphoma, with the notable exception of the *IRF4* mutation. This mutation is uncommon in germinal center B‐cell lymphomas such as diffuse large B‐cell lymphoma and classic follicular lymphoma, apart from the distinct entity of LBCL‐*IRF4* [16, 17, 18]. Of particular interest is the absence of the *MAP2K1* gene mutation seen in cases of LBCL‐*IRF4*‐R with a high‐grade follicular component, which was not detected in our case. This underscores the need for further investigation into similar cases to further explore the relationship between low‐grade follicular lymphoma or other small B‐cell lymphomas with *IRF4* rearrangement and LBCL‐*IRF4*‐R.

## AUTHOR CONTRIBUTIONS

**Ali Sakhdari**: Study design; acquisition; assembly; analysis and interpretation of data; drafting of the manuscript; critical revision of the manuscript for important intellectual content. **Jan M. A. Delabie**: Acquisition; interpretation of data; drafting of the manuscript. **Tulasi Geevar**: Acquisition and interpretation of data; drafting of the manuscript; critical revision of the manuscript for important intellectual content. **Peter J. B. Sabatini and Tong Zhang**: Acquisition; analysis, and interpretation of molecular data; drafting of the manuscript, and critical revision of the manuscript for important intellectual content. The study was approved by the “Research Ethics Board” of the University Health Network (REB#20‐5243) and conducted in compliance with the Declaration of Helsinki.

## CONFLICT OF INTEREST STATEMENT

The authors declare no conflict of interest.

## FUNDING INFORMATION

The authors received no specific funding for this work.

## ETHICS STATEMENT

This study was conducted in accordance with the Declaration of Helsinki and was approved by the University Health Network Research Ethics Board (REB approval number: 20–5243). Patient consent was waived due to the retrospective nature of the study.

## PATIENT CONSENT STATEMENT

Patient consent was waived for this study based on the University Health Network (UHN) institutional Research Ethics Board (REB) approval (CAPCR ID: 20–5243)‐permission to reproduce material from other sources.

## CLINICAL TRIAL REGISTRATION

The authors have confirmed clinical trial registration is not needed for this submission.

## Supporting information

Supporting Information

## Data Availability

The data that support the findings of this study are available from the corresponding author upon reasonable request.
