# Supplementary material for: Low‐grade follicular lymphoma with interferon regulatory factor‐4 rearrangement: Expanding the spectrum of interferon regulatory factor‐4‐rearranged lymphomas
Source: EJHaem. 2024 Aug 21;5(5):1068–71. doi: 10.1002/jha2.992 (PMC11474358; doi:10.1002/jha2.992)
Supplement: Supplementary file 1 — Supporting Information [file JHA2-5-1068-s001.docx]

Supplementary table 1: List of Genes (entire coding sequence targeted) included in Next Generation Sequencing Panel

| *ACTB* | *CD79B* | *FCRLA* | *KLHL14* | *PLCG2* | *STAT3* |
| --- | --- | --- | --- | --- | --- |
| *ACTG1* | *CD83* | *FOXC1* | *KLHL6* | *POT1* | *STAT5B* |
| *ARID1A* | *CDKN2A* | *FOXO1* | *KMT2D* | *PRDM1* | *STAT6* |
| *ATM* | *CHD2* | *GNA13* | *MAP2K1* | *PRDM15* | *TBCC* |
| *B2M* | *CIITA* | *GRHPR* | *MBTPS1* | *PRKDC* | *TBL1XR1* |
| *BCL10* | *CREBBP* | *H1-3* | *MED16* | *PRRC2A* | *TCF3* |
| *BCL2* | *CXCR4* | *H1-5* | *MEF2B* | *PRRC2C* | *TET2* |
| *BCL2L1* | *DDX3X* | *H2BC4* | *MPEG1* | *PTPN1* | *TMEM30A* |
| *BCL6* | *DNMT3A* | *HLA-A* | *MYC* | *RFTN1* | *TNFAIP3* |
| *BIRC3* | *DTX1* | *HLA-B* | *MYD88* | *RHOA* | *TNFRSF14* |
| *BRAF* | *DUSP2* | *ID3* | *NFKBIA* | *RRAGC* | *TOX* |
| *BTG1* | *DYSF* | *IDH2* | *NFKBIZ* | *S1PR2* | *TP53* |
| *BTG2* | *EDRF1* | *IL4R* | *NOL9* | *SEC24C* | *TP73* |
| *BTK* | *EIF4A2* | *INTS1* | *NOTCH1* | *SEMA4A* | *TRAF2* |
| *CARD11* | *EP300* | *IRF4* | *NOTCH2* | *SETD1B* | *UBE2A* |
| *CCND1* | *ETS1* | *IRF8* | *OSBPL10* | *SETD2* | *UBR5* |
| *CCND3* | *ETV6* | *ITPKB* | *P2RY8* | *SF3B1* | *WEE1* |
| *CD58* | *EZH2* | *JAK3* | *PIK3CG* | *SGK1* | *XBP1* |
| *CD70* | *FAS* | *JUNB* | *PIM1* | *SOCS1* | *XPO1* |
| *CD79A* | *FCGR2B* | *KLF2* | *PIM2* | *SPEN* | *ZFP36L1* |
